# A population-based cohort study for presence of ulceration among cutaneous malignant melanoma subgroups of patients

**DOI:** 10.3389/fonc.2022.929600

**Published:** 2022-11-02

**Authors:** Xinrui Li, Zichao Li, Xiaowei Yi, Xianchun Gao, Zhe Yang, Xingning Huang, Sijie Ma, Tianyuan Ma, Ziyi Deng, Lei Shang, Zhe Jian

**Affiliations:** ^1^ School of Medicine, Northwest University, Xi’an, Shaanxi, China; ^2^ Department of Health Statistics, School of Public Health, Fourth Military Medical University, Xi’an, Shaanxi, China; ^3^ Department of Burns and Cutaneous Surgery, Xijing Hospital, Fourth Military Medical University, Xi’an, Shaanxi, China; ^4^ West China School of Public Health and West China Fourth Hospital, Sichuan University, Chengdu, Sichuan, China; ^5^ College of Basic Medicine, Fourth Military Medical University, Xi’an, Shaanxi, China; ^6^ Department of Dermatology, Xijing Hospital, Fourth Military Medical University, Xi’an, Shaanxi, China

**Keywords:** cutaneous malignant melanoma, ulceration, directed acyclic graph, confounding effects, sensitivity analysis, causal inference

## Abstract

**Background:**

Observational studies suggest that ulceration is considered to be a negative prognostic factor for cutaneous melanoma. However, the impact of ulceration over different subgroups (e.g. AJCC Stage, thickness level) are controversial and its true causal effect on survival is lack of studies in the view of treating ulceration as an exposure.

**Objective:**

To explore the true causal effect of ulceration on melanoma’s survival by adopting a combination of methods to discover proper adjustment set and confirming its correctness through a variety of means.

**Methods:**

A minimal sufficient adjustment set (MSAS) was found using directed acyclic graphs (DAG) to adjust the effect of causality. Sensitivity analysis was conducted to diagnose potential confounders in addition to MSAS. Cox models were built to analyze the causality in-depth and the model was validated using a novel method. Lastly, stratified effects of ulceration were examined to illustrate its impact within subgroups.

**Results:**

Hazard ratio (HR) of ulceration after adjustment by MSAS variables was 1.99 (95% CI=1.88-2.09). The sensitivity analysis of propensity score matching and E-value both demonstrated that variables other than MSAS do not have great influence on ulceration and MSS relationship. The HR of ulceration in AJCC Stage, thickness level, invasion level and tumor extension were all monotonically decreased from 5.76 to 1.57, 4.03 to 1.78, 2.75 to 1.78 and 2.65 to 1.71 respectively.

**Conclusion:**

Ulceration in all subgroups were shown to have a significantly negative impact on MSS and its magnitude of effect was monotonically decreased as the disease progressed. The true effect of ulceration can be adjusted by MSAS and its correctness was validated through a variety of approaches.

## Introduction

Although ulceration is acknowledged as an adverse prognostic factor for cutaneous malignant melanoma (CMM) in many published literature (1–4), most studies uncovered its association relationship with melanoma-specific survival (MSS) in the aim at searching for significant factors and constructing prediction models (5–8). Some claimed that the presence of ulceration is primarily associated with certain subgroups (e.g.T2 and T3) yet not having an influence on T1 and T4 (9) whereas other articles reported no impact of ulceration in patients with tumor thickness > 2 mm (i.e. T3 and T4) (10), or its influence in overall survival (OS) is significant in all T-stages (11). The existence of conflicting results on the prognostic value of the extent of ulceration may be due to a variety of reasons. In spite of being considered as a crucial factor for melanoma survival, ulceration was never treated as an exposure to discover its true causal effect on MSS or OS. It was either used as a predictor for model development or as a stratum for comparison among subgroups. As in aforementioned situations that some unmeasured confounders might have an strength that would suffice to explain away the effect of exposure on outcome (12), ulceration is therefore ought to be treated as an exposure to diagnose in-depth its effect on melanoma survival to clarify those contradictions. In this study, we examined the role of ulceration in view of its causal relationship with MSS in a population-based cohort data sample.

## Materials and methods

### Study population

This study is based on data from the 2004 to 2015 Surveillance, Epidemiology, and End Results Program (SEER). SEER samples are chosen using SEER*Stat, version 8.3.8 (NCI, Bethesda, MD). Patients with CMM as first primary malignant tumor are eligible for this study, excluding individuals without positive histology (n=5998), survival time equals to 0 (n=10050), whose cause of death unknown (n=1790) and T stage are T0 (n=6577). A total of 165043 patients were retained for our study. Breslow thickness was categorized into four groups 1 to 4 (≤ 1mm, 1.01 to 2mm, 2.01 to 4mm, and > 4 mm), age at diagnosis was categorized into three groups “young” “middle” “old” (≤45, 45 to 60, and >60), and treatment was created based upon variables indicating surgery, chemotherapy, radiation and was categorized into five groups “surgery only”, “CT & RT w or w/o surgery”, “CT w or w/o surgery”, “RT w or w/o surgery”, “No treatment”.

Ulceration was defined as the full thickness absence of an intact epidermis above any portion of the primary tumor with an associated host reaction above the primary tumor based on histopathological examination (1).

### Causal analysis for the effect of ulceration on MSS

Variables whose missing rate less than 20% were conducted missing imputation using missForest to reduce the likelihood of residual confounding (13–15). We initiated our causal analysis by using directed acyclic graphs (DAG) (16, 17) and Pearl’s back-door criterion (16, 18) to select a minimal sufficient adjustment set (MSAS) which is accounted for confounders and potential colliding pathways to minimize the risk of biased inference (16–19). Ulceration was set as an exposure and MSS was set as an outcome. Conditional independence among variables can be statistically tested through *DAGitty*, an online tool that was built for DAG development and evaluation (http://dagitty.net) (20). The DAG-dataset consistency was checked by R package *DAGitty* that root mean square error of approximation (RMSEA) of an logistic regression was calculated for each conditional independence implied by the hypothesized DAG (19, 21). RMSEA<0.1 were considered consistent with the hypothesized DAG (22).

### Sensitivity analysis for unmeasured confounding

Sensitivity analysis was conducted to assess the robustness of causal relationship between ulceration and MSS controlled by variables in the MSAS. Our assumption is that the effect of ulceration on MSS would not be significantly affected by variables other than those in the MSAS. To evaluate this assumption, propensity score matching was used to create matched dataset of ulcerated and nonulcerated samples with MSAS variables as a baseline reference (23, 24). Odds ratio (OR) and mortality difference (MD) were calculated for comparison of the baseline estimate and estimate adjusted by potential confounder (PC). Change-in-estimate(CIE) criterion with cutoff of 10% was applied to determine if an inevitable bias on estimating causal effect of ulceration on MSS was presented (25, 26).

Cox regression model was used to estimate the effect of ulceration on MSS. Three models were built with linear predictors of ulceration only, ulceration adjusted by MSAS, and ulceration adjusted by MSAS plus other variables. They were named as model A, B, C respectively. Hazard ratio (HR) was reported in each model along with E-value, a new measure that was used for assessing how strongly an unmeasured confounder could explain away the observed effect of exposure-outcome association when determination of causality from observational studies was desired (12). (see **Supplementary Methods** for details on sensitivity analysis). The proportional hazard (PH) assumption was checked by Schoenfeld partial residuals and for the covariates violated the PH assumption, a time dependent interaction term was introduced to adjust the HR variation over time.

Based upon the result of sensitivity assessment, the effect of ulceration was further analyzed in stratified groups of crucial confounders in MSAS which showed a strong E-value compared to the E-value of ulceration itself (12).

### Model validation and evaluation

Dataset was randomly splitted to training and validation set by ratio of 2:1. Three models were developed using training set and their discrimination ability was checked by Harrell’s C-index (27). HR of ulceration in each model was presented to show the disparity of HR among adjusted and unadjusted models.

A comprehensive model validation and calibration was carried out for the model with ulceration adjusted by MSAS. Prognostic index (PI) together with Kaplan-Meier curves for ulceration and risk groups were used for model validation (28). Four risk groups were created based on 50%, 75%, 90% and 100% quantile of PI derived on training set and were validated on validation set. A stricter type of model calibration assessment was conducted in use of approximating the baseline hazard function (28). (see **Supplementary Methods** for details on method of validation).

The significance level of α=0.05 was used for all statistical tests involved in the study.

## Results

The median follow-up time is 62 months. The prognostic factors considered as an entry of DAG showed a relatively balanced percentage among categories in death of CMM but unbalanced in death of other causes or alive (**Table 1**). For instance, ulcerated patients accounted for 48% in death of CMM while only 10% otherwise. For AJCC Stage IV and Tumor extension of distant, there are 17% and 20% patients in death of CMM respectively, but both of their percentages were down to 1% otherwise. The characteristics inferred that there might be an association between the factor and death of CMM that considering them as an entry of DAG would be good to start with. Full list of characteristics of variables can be found in **Supplementary Table 2**.

**Table 1:** Characteristics of the prognostic factors as an entry of DAG.

		*n* (%) or mean (SD^1^)	
Variable	Category	Death ofCMM	Death of othersor alive
Ulceration	Yes	5640 (48%)	15880 (10%)
	No	6221 (52%)	137302 (90%)
Tumor thickness	1 (≤ 1mm)	1973 (17%)	105392 (69%)
	2 (1.01 - 2mm)	3413 (29%)	29914 (20%)
	3 (2.01 - 4mm)	2652 (22%)	10963 (7%)
	4 (> 4 mm)	3823 (32%)	6913 (5%)
AJCC Stage	I	3070 (26%)	127751 (83%)
	II	3228 (27%)	16897 (11%)
	III	3516 (30%)	7399 (5%)
	IV	2047 (17%)	1135 (1%)
Tumor extension	Localized	5480 (46%)	141763 (93%)
	Regional	4028 (34%)	9671 (6%)
	Distant	2353 (20%)	1748 (1%)
Histological subtype	Superficial spreading	1812 (15%)	50744 (33%)
	Nodular	2591 (22%)	8926 (6%)
	Lentigo	230 (2%)	9928 (6%)
	Acral lentiginous	322 (3%)	1327 (1%)
	Amelanotic	99 (1%)	471 (.3%)
	Other uncommon	6807 (57%)	81786 (53%)
Invasion level	2	620 (5%)	61471 (40%)
	3	1320 (11%)	39714 (26%)
	4	6548 (55%)	47001 (31%)
	5	3373 (28%)	4996 (3%)
Treatment	Surgery only	8658 (73%)	145230 (95%)
	CT&RT w or w/o surgery	356 (3%)	151 (0%)
	CT w or w/o surgery	1049 (9%)	1005 (1%)
	RT w or w/o surgery	818 (7%)	1034 (1%)
	No treatment	980 (8%)	5762 (4%)
Survival status	Training set	7888 (93%)	102141 (7%)
	Validation set	51041 (93%)	3973 (7%)

SD^1^, standard deviation.

For additional characteristics of prognostic factors see **Supplementary Table 1**.

**Figure 1:**
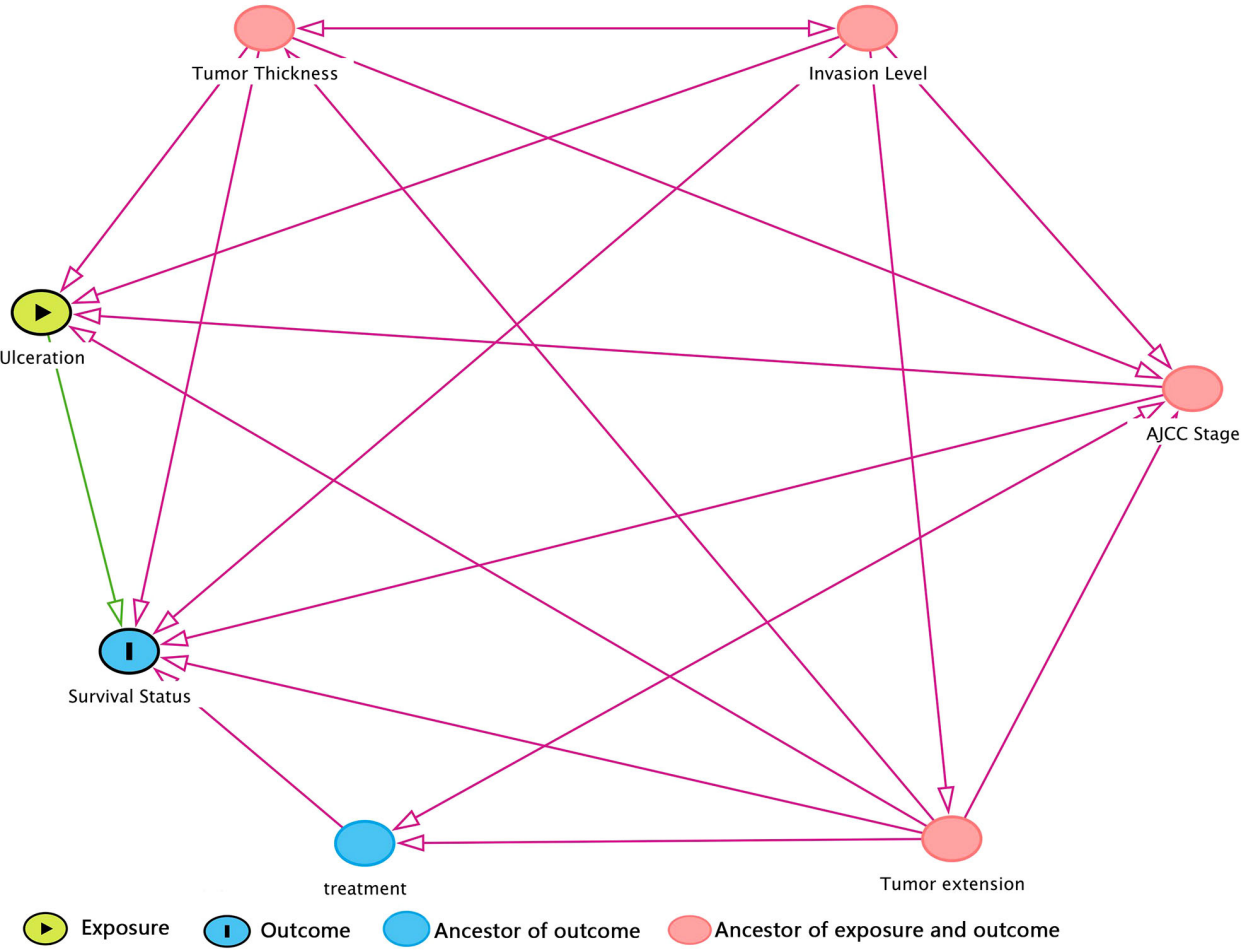
DAG (directed acyclic graph) for hypothesized causal relationship. The variables in red dot were ancestors for both exposure and outcome, which means that they were bias variables that ought to be adjusted. Hence, the minimal sufficient adjustment set (MSAS) is tumor thickness, invasion level, AJCC stage, and tumor extension. Treatment is the ancestor of outcome but not exposure, so it would not be included in MSAS.

Prior to DAG, independence test was made as a preliminary analysis for developing DAG. Detailed test results were in **Supplementary Table 3** in descending rank order of RMSEA. Variables that were unconditionally dependent of death of CMM were Tumor extension, ulceration, AJCC Stage, Invasion level, and treatment and were therefore retained to DAG development as shown in **Figure 1**. The MSAS to estimate the total effect of ulceration on death of CMM were Tumor extension, AJCC Stage, Invasion level and tumor thickness.

Based on propensity score matched data with MSAS as covariates, OR of ulcerated vs. nonulcerated was 1.41 (1.35-1.48) (**Table 2**). OR adjusted by each potential confounder was very close to 1.41 that none of them had a CIE above 10%. In fact, majority of CIE were less than 1% and most of the mortality difference were less than 3. The sensitivity assessment of potential confounders showed that all variables except for MSAS had little effect on OR of ulceration on death of CMM and therefore had no need to be adjusted.

**Table 2:** Sensitivity assessment of potential confounders by propensity score matching to evaluate the disparity of odds ratio and mortality difference for PC-unadjusted and PC-adjusted results.

Potential confounder (PC)	PC-adjustedOR^1^ of exposure-outcome (95% CI)	CIE^2^	PC-adjusted MD^3^(per 1000)	PC-adjustedMD bias(per 1000)	P-value for MD
None	1.41 (1.35-1.48)	–	61.04	0	<0.0001
Age	1.44 (1.37-1.51)	1.85%	64.02	2.98	<0.0001
Sex	1.41 (1.35-1.48)	0.00%	61.10	0.06	<0.0001
Histological subtype	1.37 (1.31-1.43)	3.10%	55.84	-5.20	<0.0001
Treatment	1.42 (1.36-1.48)	0.46%	61.68	0.64	<0.0001
UV exposure	1.41 (1.35-1.48)	0.14%	61.23	0.19	<0.0001
Recurrence	1.41 (1.35-1.48)	0.00%	61.04	0	<0.0001
laterality	1.41 (1.35-1.48)	0.00%	60.95	-0.09	<0.0001
site	1.46 (1.40-1.53)	3.60%	66.74	5.70	<0.0001
Marriage	1.41 (1.35-1.48)	0.00%	61.06	0.02	<0.0001
Race	1.41 (1.35-1.47)	0.26%	60.66	-0.38	<0.0001
RLN examined	1.41 (1.35-1.47)	0.29%	60.57	-0.47	<0.0001
SLNB	1.41 (1.35-1.48)	0.00%	61.10	0.06	<0.0001

OR^1^, odds ratio; CIE^2^, Change-in-estimate; MD^3^, mortality difference.

The cox regression models continually showed consistency with causal analysis above. The HR of ulcerated patients in Model B (1.99, 1.88-2.09) and C (2.05, 1.94-2.17) were drastically adjusted compared to Model A (7.98, 7.63-8.34) whereas the overlapping of 95% CI of HR in Model B and C implied that these two models did not make significant change on the estimates (**Table 3**). In addition to that, Harrell’s C-index of the latter two models (Model B: 0.89, se=0.002; Model C: 0.90, se=0.002), were very close and both were considerably more accurate than C-index in Model A (0.7, se=0.003). The result came to conclusion that MSAS variables played an indispensable role in model development in terms of discovering true effect of ulceration on MSS as well as maintaining a high level of model accuracy. Meanwhile, variables other than MSAS seem to be redundant in ulceration-MSS causality analysis and model development.

**Table 3:** Hazard ratios and discrimination measures for three models developed in training set.

		Model A^1^	Model B^2^	Model C^3^
Ulceration	Yes/No	7.98	1.99	2.05
	95% CI	(7.63, 8.34)	(1.88, 2.09)	(1.94, 2.17)
	P-value	<0.0001	<0.0001	<0.0001
Harrell’s C-index (SE)		0.7 (0.003)	0.887 (0.002)	0.902 (0.002)

Model A^1^ = COX model with only ulceration as a linear predictor.

Model B^2^ = COX model with ulceration and MSAS variables as linear predictors.

Model C^3^ = COX model with all candidate variables as linear predictors.

The full list of HR for three models was shown in **Supplementary Table 5**. E-value as a measure of confounding influence was reported along with HR for each categorical variable. In model B, E-value for ulcerated patients was 3.39 (LL CI=3.17). Use this as a benchmark to assess other variables, we can observe that most MSAS variables had E-value higher than 3.39 and thus were worth to be used as a stratum to further examine the effect of ulceration on MSS. By controlling for rest of MSAS variables, HRs of ulcerated patients in each stratum had worse prognosis and their descending trend indicated that the effect of ulceration on MSS were stronger in earlier phase of disease (P<0.0001) (**Table 4**). In model C, all variables other than MSAS had E-value close to 1, which means that they would make trivial influence to explain away the effect of ulceration on MSS (**Supplementary Table 5**).

**Table 4:** Stratified HR of ulceration adjusted by rest of MSAS variables.

		HR of ulceration (95% CI)	P-value
Invasion level: 2		2.75 (1.83-4.14)	<0.0001
Invasion level: 3		2.17 (1.79-2.64)	<0.0001
Invasion level: 4		1.92 (1.79-2.06)	<0.0001
Invasion level: 5		1.78 (1.63-1.95)	<0.0001
AJCC Stage: I		5.76 (4.92-6.74)	<0.0001
AJCC Stage: II		2.03 (1.84-2.23)	<0.0001
AJCC Stage: III		1.84 (1.68-2.01)	<0.0001
AJCC Stage: IV		1.57 (1.40-1.77)	<0.0001
Thickness: 1 (≤ 1mm)		4.03 (3.50-4.64)	<0.0001
Thickness: 2 (1.01 - 2mm)		2.06 (1.79-2.37)	<0.0001
Thickness: 3 (2.01 - 4mm)		1.78 (1.62-1.96)	<0.0001
Thickness: 4 (> 4 mm)		1.78 (1.63-1.94)	<0.0001
Tumor extension: localized		2.65 (2.40-2.93)	<0.0001
Tumor extension: regional		1.88 (1.74-2.04)	<0.0001
Tumor extension: distant		1.71 (1.53-1.91)	<0.0001
AJCC Stage I&II	Thickness:1	5.33 (4.53-6.26)	<0.0001
AJCC Stage I&II	Thickness:2	1.86 (1.64-2.11)	<0.0001
AJCC Stage I&II	Thickness:3	1.94 (1.71-2.21)	<0.0001
AJCC Stage I&II	Thickness:4	2.08 (1.80-2.41)	<0.0001

**Figure 2:**
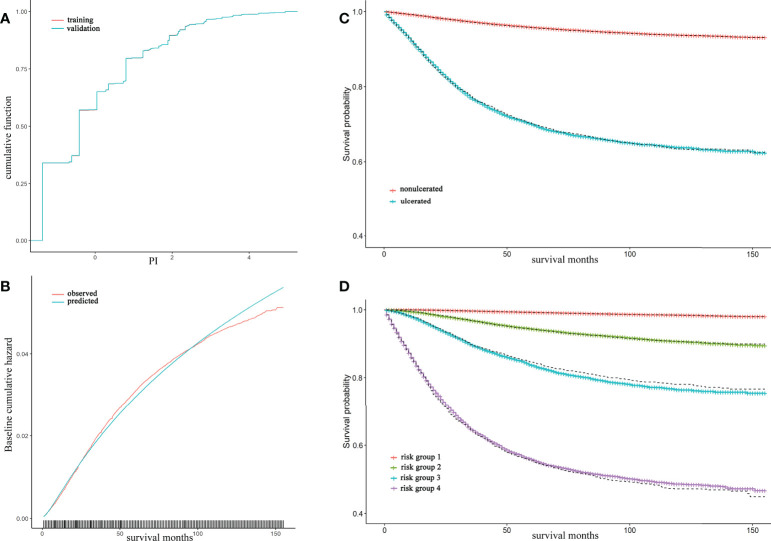
Model B validation and calibration, together with Kaplan-Meier (KM) curve of ulceration and risk groups The cumulative distribution of prognostic index (PI) for training and validation set were almost coincident and can hardly be distinguished through eyes **(A)**; The observed and predicted baseline hazard function in training set showed a well calibrated ability of model B **(B)**; The red and blue lines were accounted for nonulcerated and ulcerated KM and were estimated in training set whereas the dash lines were estimated survival curve in validation set **(C)**; The solid lines from top to bottom represented risk group 1 to 4 by 50%, 75%, 90%, 100% quantile of PI respectively. Dashed lines were their estimated survival curve in validation set **(D)**.

Model validation was conducted for model B in three means. The cumulative PI of training and validation set was nearly to coincide that the difference was hard to tell visually (**Figure 2A**). The observed and predicted baseline hazard function on training set showed a good calibrated ability of model B over survival time (**Figure 2B**). Both Kaplan-Meier curves of ulceration and risk groups exhibited that the estimated survival probability in validation set was coincident with the survival curves in training set (**Figures 2C, D**).

## Discussion

We confirmed that ulceration had an overall adverse effect on MSS and found an appropriate MSAS that can be used to adjust the effect of ulceration on MSS. After adjustment by MSAS variables (e.g. AJCC Stage, Invasion level, Tumor thickness, Tumor extension), the HR of ulcerated patients was 1.99 (1.88-2.09). Furthermore, taking crucial confounders in MSAS as strata to analyze the effect of ulceration on MSS, we found that ulceration played an adverse role in all stratified groups (all p<0.0001) and its descending trend indicates that the magnitude of its effect was weakened as the disease progressed (**Table 4**).

Specifically, the HR of ulceration in Invasion level was from 2.75 to 1.78, in AJCC Stage it was from 5.76 to 1.57, in tumor thickness it was from 4.03 to 1.78, in tumor extension it was from 2.65 to 1.71, and given Stage I&II, in tumor thickness it was from 5.33 to 2.08 (**Table 4**). In a recent study of impact of ulceration in stage I&II Italian Melanoma Intergroup, Portelli et al. (10) stated that ulceration only had a significant impact for tumor thickness ≤ 2mm, but no impact for tumor thickness >2mm. This inconsistency with our finding could be due to reasons: (i) small sample data (ii) estimates on thickness can be doubted. The paper reported HR of thickness increase per mm greater than 1 in univariate analysis yet less than 1 in multivariate analysis. As far as we know, no evidence support the idea that the increase of tumor thickness could result a better prognosis in any circumstances (6, 7, 29). (iii) lack of an appropriate adjustment set for controlling the crucial confounders. The variables in COX regression model were selected based on AIC (Akaike Information Criterion), which was primarily used to assess the goodness of fit of a model in terms of its prediction error (30, 31). Thus, by using this means of variable selection, the primary goal was to find better fitted model, rather than discovering the true impact of a factor on an outcome. In contrary, our result showed that given Stage I&II, the risk of death for ulcerated patients was even higher in thickness 4 (HR=2.08) than in thickness 2 (HR=1.86) or 3 (HR=1.94). In Eigentler et al. (9) article, he reported that ulceration had a negative impact on prognosis in T2 and T3 but not in T1 and T4. By adjusting age and histological subtype, he reported a relative risk of 1.2 for ulceration in Stage III and further claimed that ulceration should not be the focus in this stage. This underestimation of ulceration’s impact could due to the reasons expounded above that appropriate confounders might not be given to adjust the effect properly. Besides from that, the new AJCC staging system also did not consider ulceration as an independent prognostic factor in Stage III for lack of research evidence to conclude its significant impact on Stage III or IV. Interestingly, a most recent study made similar conclusion as ours that ulceration had significant impact in all stages and the cause of those contradictions were elaborated in the article (11).

In analyzing the causality of ulceration on MSS, we conducted a combination of methods to discover proper adjustment set and confirmed its correctness through a variety of means. Our study can be summarized in six steps: in step one, DAG as a simple graphical tool for delineating the exposure-outcome causal relationship was commonly recognized by epidemiologists and would be a good set to start with for our analysis (16, 32, 33). However, it can be relatively arbitrary to construct and select MSAS solely depend on DAG. Hence, in step two we conducted sensitivity analysis using propensity score matching to estimate OR and MD to assess if those potential confounders which were not included in MSAS could lead to a strong bias on the effect of ulceration. The result showed no major change (> 10%) in OR between PC-unadjusted and -adjusted (**Table 2**). Therefore, it was more confident for us to put this set of variables along with ulceration into model building. In step three, the COX regression model was built in three ways: ulceration only, ulceration with MSAS, ulceration with all variables; namely, model A, B and C. In comparison of model’s discrimination and HR of ulceration, Harrell’s C-index showed that model B (C-index=0.89, se=0.002) and C (C-index=0.90, se=0.002) outperformed model A (C-index=0.70, se=0.003). Moreover, given a lot more variables added, model C did not elevate the accuracy too much so that variables other than MSAS seem to be redundant. HR of ulceration was drastically adjusted from model A (7.98, 7.63-8.34) to model B (1.99, 1.88-2.09) and C (2.05, 1.94-2.17) but no significant change between model B and C also suggest that those additional variables in model C might not be necessary for adjusting effect of ulceration on risk of death by CMM. Furthermore, E-value was used to evaluate the confounding effect in each category of variables. A clear distinction in E-value was found between MSAS variables and others (**Supplementary Table 5**) that E-value of most MSAS variables were greater than ulceration (3.39, LL CI=3.17) but others were close to 1. According to VanderWeele et al. (12), a E-value of 1 means no confounding effect at all. This comprehensive assessment of confounding effects combined by COX regression model strongly demonstrated that our way of exploring causality between ulceration and MSS can be sufficiently supported. In step five, the model B was validated using a novel method. By comparing results from training and validation set, the cumulative PI and survival curves of ulceration and risk groups were shown great consistency. Furthermore, with predicted baseline hazard function, we were able to calibrate how good the model really was in each slot of time by comparing it to the observed baseline hazard function (28). This way of measuring calibration is stricter and more recommended as opposed to log-rank test, for which its *P*-value does not quantify discrimination. At last, stratified HR of ulceration was estimated and helped to explain the confusions and conflicts in the study of ulceration’s impact on prognosis.

### Limitations

Although the data collected from SEER database includes as many variables as possible, some important variables such as tumor size has missing data more than 50% so we have to exclude those variables for modeling. As an exposure, ulceration could have several statuses in terms of its extent, percentage, and type. In this study, only the presence of ulceration was available in the database. The study is lack of external validation using datasets from different sources and this will be our next step in the future.

## Conclusions

In the process of DAG construction, we found that treatment was not associated with factors like Invasion level, Tumor thickness and ulceration given certain conditions (**Supplementary Table 4**). This inferred that once the extension of disease is decided, Invasion level and tumor thickness would be hardly shifted by means of treatment. Ulceration is also independent of treatment once tumor extension, AJCC Stage, Invasion level and tumor thickness are given.

In conclusion, our work confirmed causality between ulceration and MSS, and examined the magnitude of their causal relationship through a variety of thoughtful approaches and concluded that the effects of ulceration on prognosis were descended as the disease progressed.

## Data availability statement

The datasets presented in this study can be found in online repositories. The names of the repository/repositories and accession number(s) can be found below: https://seer.cancer.gov/.

## Ethics statement

The studies involving human participants were reviewed and approved by National Cancer Institute, USA. The patients/participants provided their written informed consent to participate in this study.

## Author contributions

The original design, draft writing, data analysis were conducted by XL, partially by ZL; the data collection, data processing was conducted by XY, XH, SM, TM; the table and figure preparation was conducted by ZY, ZD; the manuscript editing was conducted by XG; the proofreading, clinical implications was confirmed and revised by LS, ZJ. All authors contributed to the article and approved the submitted version.

## Funding

This work was financially supported by the National Natural Science Foundation of China. (No.82173627).

## Acknowledgments

The authors obtained authorization to exact and analyze the research data stored in the SEER program from the National Cancer Institute, USA. Informed patient consent was not required to access data through the SEER database. This study was conducted in strict accordance with the 1964 Helsinki Declaration and subsequent amendments or similar ethical standards.

## Conflict of interest

The authors declare that the research was conducted in the absence of any commercial or financial relationships that could be construed as a potential conflict of interest.

## Publisher’s note

All claims expressed in this article are solely those of the authors and do not necessarily represent those of their affiliated organizations, or those of the publisher, the editors and the reviewers. Any product that may be evaluated in this article, or claim that may be made by its manufacturer, is not guaranteed or endorsed by the publisher.
