# Supplementary material for: A population-based cohort study for presence of ulceration among cutaneous malignant melanoma subgroups of patients
Source: Front Oncol. 2022 Nov 2;12:929600. doi: 10.3389/fonc.2022.929600 (PMC9666370; doi:10.3389/fonc.2022.929600)
Supplement: Supplementary file 1 [file DataSheet_1.pdf]

Supplementary Methods

Missing Imputation Approach

Missing data are a common problem that usually showed up in large-scale cohort data acquisition from real world. For observational studies that include a high level of dimensions, taking missing observations out of the study could result in a loss of statistical power or lead to an increase of selection bias[(1)]. Conventional missing imputation technologies are widely known to have certain limitations such as: (i) some methods can be solely used for continuous variables (e.g. *k* nearest neighbours[(2)]) whereas some are only for categorical variables (e.g. saturated multinomial model[(3)]). Such methods fail to handle mixed-type of data simultaneously so that the efficacy and convenience of missing imputation can be limited. (ii) mostly, methods such as multivariate imputation by chained equations (MICE)[(4)] could handle mixed-type of data but it depends on tuning parameters or models with a prior knowledge for the sample structures. Moreover, all these parametric methods require assumptions such as normality, linearity, and independence of variables, which are often violated from real world dataset. missForest as a non-parametric, random forest based imputation method was recently being approved to have better performance than those conventional methods and it allows any type of input data to make as few as possible assumptions about data structure for missing values ranging from 10% to 30%[(5)]. The procedure is that each variable with missing values would be treated as a predicted outcome to process random forest based on other variables and the out-of-bag error can be seen as an evaluation for imputation performance.

Sensitivity analysis

Sensitivity analysis was conducted for the conditional independence assumption that no adjustment other than MSAS variables are needed in order to have an un-confounded estimate of the effect of ulceration on MSS. The assumption was firstly tested based on a propensity score matching estimator[6-8]. With MSAS variables as predictor variables, a logistic regression model was fitted to get a predicted probability of death of CMM for each individual as a propensity score. And then the ulcerated individuals were matched with nonulcerated individuals whose propensity score were lied within ± 0.001 [9,10]. The estimates of odds ratio (OR) and mortality difference (MD) were calculated based on the data matrix with match ulcerated and nonulcerated individuals. Variables other than MSAS variables were seen as potential confounders (PC) and each time one variable was added up with MSAS variables as predictors in the logistic regression model to fit and get propensity score so that new matched dataset could be generated. The estimates of PC-adjusted OR and MD were hence calculated in order to evaluate the Change-in-estimate (CIE). CIE was simply the percentage difference between PC-adjusted and PC-unadjusted OR, which can be written as: $\mathrm{CIE} = \frac{|\mathrm{OR}_{\mathrm{pc-adjusted}} - \mathrm{OR}_{\mathrm{pc-unadjusted}}|}{\mathrm{OR}_{\mathrm{pc-unadjusted}}} \times 100\%$. The absolute bias of MD was also reported with CIE.

Furthermore, E-value as a measure of confounding effect was estimated along with hazard ratio (HR) in COX model. According to VanderWeele et.al.[11], E-value is the minimum strength of association that an unmeasured confounder would need to have with both the treatment and outcome, conditional on the measured covariates, to fully

explain away a specific treatment (or exposure)-outcome association. It focuses on the magnitude of the confounder associations that could produce confounding bias equal to observed treatment (or exposure)-outcome association. For an observed HR of ulceration on MSS, the calculation of E-value can be written as:

$$\mathrm{E-value} = \mathrm{HR} + \sqrt{\mathrm{HR} \times (\mathrm{HR} - 1)}.$$

In terms of the confidence interval (CI) of E-value, the criterion for calculation is as below:

(i) when HR>1, only the lower limit (LL) will be calculated, and it can be written as:

$$\text{If } \mathrm{LL_{HR}} \leq 1, \text{then } \mathrm{LL_{E-value}} = 1,$$

$$\text{else if } \mathrm{LL_{HR}} > 1, \text{then } \mathrm{LL_{E-value}} = \mathrm{LL_{HR}} + \sqrt{\mathrm{LL_{HR}} \times (\mathrm{LL_{HR}} - 1)}\ ;$$

(ii) when HR<1, only the upper limit (UL) will be calculated, and it can be written as:

$$\text{Let } HR^* = 1/HR, E - value = HR^* + \sqrt{HR^* \times (HR^* - 1)}$$

$$\text{If } \mathrm{UL_{HR}} \geq 1, \text{then } \mathrm{UL_{E-value}} = 1,$$

$$\text{else if } \mathrm{UL_{HR}} < 1, \text{then } \mathrm{UL_{E-value}} = 1/\mathrm{UL_{HR}} + \sqrt{1/\mathrm{UL_{HR}} \times (1/\mathrm{UL_{HR}} - 1)}.$$

Model validation

The COX model with MSAS as predictors was validated using a novel method. Three levels of information were used to validate the model. (i)L1: Prognostic Index (PI), (ii) L2: Kaplan-Meier curves for ulceration and risk groups, (iii) L3: predicted baseline hazard function[(12)]. In L1, PI can be defined as the weighted sum of variables where the weights are the regression coefficients. In this case, linear predictors are reduced to one dimension, e.g. PI for each individual. In L2, risk groups were defined

as 50%, 75%, 90% and 100% quantile of PI derived on training set and were validated on validation set. According to Royston et.al., in L3, fractional polynomial regression was used to approximate the log baseline cumulative hazard function, $lnH_0(t)$ as a smooth function of time in the training set. With ordinary least square estimation, we can obtain function as below:

$$lnH_0(t) = -1.28 - 6.47t^{-0.5} - 2.67t^{-0.5}lnt$$

Predicted hazard baseline function is hence: $exp(lnH_0(t))$.

Supplementary Tables

Supplementary Table 1. Comparison of the number and percentage of variables before and after data imputation

Variable	Category	Missing rate	Before imputation		After imputation	
			n (%)		*n* (%)	
Tumor thickness	1	7.64%	106710	69.8%	107365	65.1%
	2		23100	15.1%	33327	20.2%
	3		13271	8.7%	13615	8.2%
	4		9738	6.4%	10736	6.5%
Ulceration	No	6.60%	134245	87.1%	143523	87.0%
	Yes		19963	12.9%	21520	13.0%
AJCC Stage	I	9.66%	116701	78.1%	130821	79.3%
	II		18885	12.6%	20125	12.2%
	III		10638	7.1%	10915	6.6%
	IV		3182	2.1%	3182	1.9%
Tumor extension	Localized		141925	88.9%	147243	89.2%
	Regional		13683	8.6%	13699	8.3%
	Distant	3.35%	4037	2.5%	4101	2.5%
Invasion level	2	19.57%	51227	38.4%	62091	37.6%
	3		34081	25.6%	41034	24.9%
	4		42272	31.7%	53549	32.4%
	5		5683	4.3%	8369	5.1%
Marriage	Married	25.67%	102207	83.2%	130510	79.1%
	Unmarried		20703	16.8%	34533	20.9%
Race	White		154865	98.6%	162154	98.2%
	Black		757	0.5%	826	0.5%
	Asian or Pacific Islander		1053	0.7%	1371	0.8%
	Indian/Alaska Native	4.69%	353	0.2%	692	0.4%
RLN[1] examined	Yes		57138	35.3%	57397	34.8%
	No	2.02%	104661	64.7%	107646	65.2%
SLNB[2]	yes		43421	26.6%	43616	26.4%
	no	1.15%	119744	73.4%	121427	73.6%

Applicable variables with missing values were imputed by the method of missForest, a random forest based algorithm that has been acknowledged to have stronger missing imputation ability than MCIE or other common mechanism.

Most variables maintained roughly equal percentage in each category, Marriage and Invasion level had a relatively high missing rate, which lead to slightly discrepancy between imputed and non-imputed data.

RLN[1]= Regional lymph nodes

SLNB[2]= Sentinel lymph nodes biopsy

Supplementary Table 2. Full list of characteristics of variables in this study

Variable	Category	*n* (%) or mean (SD)	
		**Died caused by CMM**	**Died caused by others or alive**
Sex	Female	3772 (32%)	68902 (45%)
	Male	8089 (68%)	84280 (55%)
Age	Young (age≤45)	1606 (14%)	33633 (22%)
	Middle (45<age≤60)	3332 (28%)	49310 (32%)
	Old (age>60)	6923 (58%)	70239 (46%)
Race	White	11468 (97%)	150686 (98%)
	Black	167 (1%)	659 (.4%)
	Asian or Pacific Islander	180 (2%)	1191 (1%)
	Indian/Alaska Native	46 (.4%)	646 (.4%)
Marriage	Married	9661 (81%)	120849 (79%)
	Unmarried	2200 (19%)	32333 (21%)
Tumor thickness	1	1973 (17%)	105392 (69%)
	2	3413 (29%)	29914 (20%)
	3	2652 (22%)	10963 (7%)
	4	3823 (32%)	6913 (5%)
AJCC Stage	I	3070 (26%)	127751 (83%)
	II	3228 (27%)	16897 (11%)
	III	3516 (30%)	7399 (5%)
	IV	2047 (17%)	1135 (1%)
Tumor extension	Localized	5480 (46%)	141763 (93%)
	Regional	4028 (34%)	9671 (6%)
	Distant	2353 (20%)	1748 (1%)
Histological subtype	Superficial spreading	1812 (15%)	50744 (33%)
	Nodular	2591 (22%)	8926 (6%)
	Lentigo	230 (2%)	9928 (6%)
	Acral lentiginous	322 (3%)	1327 (1%)
	Amelanotic	99 (1%)	471 (.3%)
	Other uncommon	6807 (57%)	81786 (53%)
Ulceration	Yes	5640 (48%)	15880 (10%)
	No	6221 (52%)	137302 (90%)
Invasion level	2	620 (5%)	61471 (40%)
	3	1320 (11%)	39714 (26%)
	4	6548 (55%)	47001 (31%)
	5	3373 (28%)	4996 (3%)
Treatment	Surgery only	8658 (73%)	145230 (95%)
	CT&RT w or w/o surgery	356 (3%)	151 (0%)

	CT w or w/o surgery	1049 (9%)	1005 (1%)
	RT w or w/o surgery	818 (7%)	1034 (1%)
	No treatment	980 (8%)	5762 (4%)
Recurrence	Yes	374 (3%)	1985 (1%)
	No	11487 (97%)	151197 (99%)
Laterality	One side	10965 (92%)	140453 (92%)
	Paired sides	896 (8%)	12729 (8%)
Tumor site	Upper limb and shoulder	2119 (18%)	40764 (27%)
	Head/neck	2933 (25%)	29641 (19%)
	Lower limb and hip	2129 (18%)	28811 (19%)
	Overlapping	10 (.08%)	122 (.08%)
	Trunk	3649 (31%)	52458 (34%)
	Others	1021 (9%)	1386 (1%)
RLN[1] examined	Yes	7099 (60%)	50298 (33%)
	No	4762 (40%)	102884 (67%)
SLNB[2]	Yes	4028 (34%)	39588 (26%)
	No	7833 (66%)	113594 (74%)
UV exposure	High	8306 (70%)	106023 (69%)
	Low	3555 (30%)	47159 (31%)

The statistical summary of variables extracted from SEER database was exhibited to provide a ground level conception of variable distribution.
RLN[1]= Regional lymph nodes
SLNB[2]= Sentinel lymph nodes biopsy.

Supplementary Table 3. Test of independence among candidate variables in the entry of DAG

	RMSEA	df	p-value	rmsea 2.5%	rmsea 97.5%
AJCS[1] _||_ ExoD[2]	0.4983	6	<0.0001	0.5033	0.5033
AJCS _||_ Ulcr[3]	0.3513	3	<0.0001	0.3485	0.3541
ExoD _||_ SrvS[4]	0.2927	2	<0.0001	0.2893	0.2961
AJCS _||_ TmrT[5]	0.2914	9	<0.0001	0.2898	0.2930
TmrT _||_ Ulcr	0.2905	3	<0.0001	0.2877	0.2933
ExoD _||_ InvL[6]	0.2881	6	<0.0001	0.2861	0.2900
SrvS _||_ Ulcr	0.2852	1	<0.0001	0.2804	0.2900
AJCS _||_ SrvS	0.2573	3	<0.0001	0.2545	0.2601
InvL _||_ TmrT	0.2558	9	<0.0001	0.2542	0.2574
ExoD _||_ Ulcr	0.2348	2	<0.0001	0.2314	0.2382
InvL _||_ Ulcr	0.2280	3	<0.0001	0.2252	0.2308
AJCS _||_ InvL	0.2273	9	<0.0001	0.2257	0.2289
ExoD _||_ TmrT	0.2126	6	<0.0001	0.2106	0.2146
SrvS _||_ TmrT	0.2067	3	<0.0001	0.2039	0.2095
InvL _||_ SrvS	0.2026	3	<0.0001	0.1999	0.2054
ExoD _||_ trtm[7]	0.1641	8	<0.0001	0.1624	0.1658
SrvS _||_ trtm	0.1448	4	<0.0001	0.1425	0.1473
HstS _||_ Ulcr	0.1430	5	<0.0001	0.1408	0.1451
AJCS _||_ trtm	0.1402	12	<0.0001	0.1388	0.1416
HstS[8] _||_ TmrT	0.1116	15	<0.0001	0.1104	0.1129
AJCS _||_ HstS	0.1040	15	<0.0001	0.1028	0.1053
InvL _||_ trtm	0.0907	12	<0.0001	0.0893	0.0921
HstS _||_ InvL	0.0880	15	<0.0001	0.0868	0.0893
HstS _||_ SrvS	0.0853	5	<0.0001	0.0832	0.0875
ExoD _||_ HstS	0.0813	10	<0.0001	0.0798	0.0828
Ulcr _||_ trtm	0.0770	4	<0.0001	0.0746	0.0794
TmrT _||_ trtm	0.0738	12	<0.0001	0.0724	0.0752
Age _||_ Ulcr	0.0678	2	<0.0001	0.0644	0.0712
HstS _||_ Age	0.0574	10	<0.0001	0.0560	0.0590
AJCS _||_ Age	0.0527	6	<0.0001	0.0508	0.0547
SrvS _||_ Age	0.0489	2	<0.0001	0.0455	0.0524
TmrT _||_ Age	0.0466	6	<0.0001	0.0447	0.0487
InvL _||_ Age	0.0403	6	<0.0001	0.0384	0.0423
HstS _||_ trtm	0.0278	20	<0.0001	0.0268	0.0289
UVex[9] _||_ trtm	0.0276	4	<0.0001	0.0253	0.0301
HstS _||_ UVex	0.0224	5	<0.0001	0.0203	0.0247
InvL _||_ UVex	0.0181	3	<0.0001	0.0154	0.0210
Age _||_ trtm	0.0163	8	<0.0001	0.0147	0.0182
ExoD _||_ Age	0.0162	4	<0.0001	0.0139	0.0187

UVex _||_ Age	0.0080	2	<0.0001	0.0046	0.0117
UVex _||_ Ulcr	0.0068	1	0.0034	0.0000	0.0118
ExoD _||_ UVex	0.0066	2	0.0003	0.0030	0.0103
SrvS _||_ UVex	0.0038	1	0.0641	0.0000	0.0091
AJCS _||_ UVex	0.0020	3	0.1773	0.0000	0.0059

Independence test was conducted to construct DAG. RMSEA>0.1 was considered to be violated the hypothesis of independence between the two variable either with or without conditional variables[(13)].

The table was ranked by descending order of RMSEA. Variables associated with death of CMM were Tumor extension, Ulceration, AJCC Stage, Tumor Thickness, Invasion level, and treatment.

AJCS[1]= AJCC Stage; ExoD[2]= Tumor extension; Ulcr[3]= Ulceration; SrvS[4]= Survival Status; TmrT[5]= Tumor thickness; InvL[6]= Invasion level; trtm[7]= treatment; HstS[8]= Histological subtype; Uvex[9]= UV exposure;

Supplementary Table 4. DAG dataset test of consistency

	RMSEA[1]	df	p-value	rmsea 2.5%	rmsea 97.5%
Invasion level _‖_ Treatment | Tumor extension	0.0482	32	<0.0001	0.0437	0.0542
Tumor thickness _‖_ Treatment | Tumor extension	0.0490	36	<0.0001	0.0447	0.0549
Ulceration _‖_ Treatment | Tumor extension, AJCC Stage, Invasion level, Tumor thickness	0.0874	204	<0.0001	0.0107	0.4054

The consistency table reflects the relationship among exposure, outcome and other variables shown in DAG. This result indicates that given a Tumor extension (e.g. localized, regional, distant), different types of treatment do not have significant difference on tumor's invasion level and depth of thickness. The state of ulceration was also independent of treatment, conditional on level of Tumor extension, AJCC Stage, Invasion level, and tumor thickness.

RMSEA[1]= Root mean-square error of approximation.

Supplementary Table 5. Hazard Ratio and E-value for three models developed using training set

Model	Variable	HR (95% CI)	P-value	E-value (LL[1] CI)
**Model A**	Uleration			
	No	Reference		
	Yes	7.98 (7.63-8.34)	<0.0001	15.44 (14.74)
**Model B**	Ulceration			
	No	Reference		
	Yes	1.99 (1.88-2.09)	<0.0001	3.39 (3.17)
	Tumor extension			
	Distant	Reference		
	Localized	0.64 (0.53-0.77)	<0.0001	2.5 (1.93)
	Regional	0.78 (0.67-0.9)	0.0006	1.89 (1.47)
	Invasion level			
	2	Reference		
	3	2.6 (2.3-2.93)	<0.0001	4.63 (4.04)
	4	4.09 (3.64-4.6)	<0.0001	7.65 (6.74)
	5	5.45 (4.75-6.26)	<0.0001	10.38 (8.97)
	Tumor Thickness			
	1	Reference		
	2	2.13 (1.96-2.32)	<0.0001	3.68 (3.33)
	3	2.21 (1.99-2.45)	<0.0001	3.85 (3.4)
	4	3.33 (3.01-3.69)	<0.0001	6.13 (5.47)
	AJCC Stage			
	I	Reference		
	II	1.5 (1.36-1.64)	<0.0001	2.36 (2.06)
	III	3.19 (2.78-3.66)	<0.0001	5.83 (5.01)
	IV	9.63 (7.96-11.65)	<0.0001	18.75 (15.4)
**Model C**	Ulceration			
	No	Reference		
	Yes	2.05 (1.94-2.17)	<0.0001	3.52 (3.3)
	Tumor extension			
	Distant	Reference		
	Localized	0.6 (0.5-0.72)	<0.0001	2.73 (2.12)
	Regional	0.79 (0.68-0.91)	0.0011	1.85 (1.43)
	Invasion level			
	2	Reference		
	3	2.87 (2.54-3.23)	<0.0001	5.18 (4.52)

4	4.57 (4.05-5.15)	<0.0001	8.61 (7.57)
5	4.86 (4.22-5.59)	<0.0001	9.19 (7.92)
Tumor Thickness			
1	Reference		
2	2.1 (1.93-2.29)	<0.0001	3.62 (3.27)
3	2.04 (1.84-2.27)	<0.0001	3.51 (3.08)
4	2.99 (2.7-3.32)	<0.0001	5.43 (4.83)
AJCC Stage			
I	Reference		
II	1.43 (1.3-1.57)	<0.0001	2.21 (1.92)
III	3.03 (2.63-3.49)	<0.0001	5.5 (4.69)
IV	5.46 (4.47-6.66)	<0.0001	10.39 (8.42)
Sex			
Female	Reference		
Male	1.29 (1.23-1.36)	<0.0001	1.91 (1.76)
Age			
Middle (45<age ≤ 60)	Reference		
Old (age>60)	1.37 (1.3-1.45)	<0.0001	2.09 (1.93)
Young (age≤45)	0.76 (0.71-0.82)	<0.0001	1.96 (1.74)
Treatment			
CT&RT w or w/o surgery	Reference		
CT w or w/o surgery	0.76 (0.65-0.89)	0.0005	1.96 (1.51)
No treatment	0.78 (0.67-0.92)	0.0023	1.88 (1.41)
RT w or w/o surgery	0.67 (0.57-0.79)	<0.0001	2.33 (1.84)
Surgery only	0.4 (0.34-0.46)	<0.0001	4.48 (3.79)
Histological Subtype			
Acral lentiginous	Reference		
Amelanotic	0.91 (0.69-1.21)	0.5322	1.41 (1)
Lentigo	0.54 (0.44-0.68)	<0.0001	3.08 (2.3)
Nodular	0.91 (0.78-1.06)	0.2076	1.44 (1)
Other uncommon	0.78 (0.68-0.91)	0.0012	1.87 (1.43)
Superficial spreading	0.64 (0.55-0.75)	<0.0001	2.48 (1.99)
UV exposure			
High	Reference		
Low	0.99 (0.94-1.04)	0.6611	1.12 (1)
Recurrence			
No	Reference		
Yes	1.55 (1.37-1.76)	<0.0001	2.48 (2.08)
Laterality			
One side	Reference		

Paired sides	0.98 (0.89-1.07)	0.5888	1.18 (1)
Site			
Head/neck	Reference		
Lower limb and hip	0.78 (0.72-0.84)	<0.0001	1.9 (1.68)
Overlapping	0.86 (0.41-1.8)	0.6799	1.61 (1)
Others	1.11 (0.99-1.24)	0.0627	1.47 (1)
Trunk	0.89 (0.83-0.95)	0.0004	1.5 (1.29)
Upper limb and shoulder	0.68 (0.63-0.73)	<0.0001	2.3 (2.08)
Marriage			
Married	Reference		
Unmarried	1.05 (0.99-1.12)	0.0768	1.29 (1)
Race			
Indian/Alaska Native	Reference		
Asian or Pacific Islander	0.95 (0.65-1.4)	0.8095	1.27 (1)
Black	0.89 (0.6-1.32)	0.5685	1.49 (1)
White	0.79 (0.56-1.11)	0.1747	1.84 (1)
RLN examined			
No	Reference		
Yes	0.83 (0.77-0.88)	0.0001	1.71 (1.52)
SLNB			
No	Reference		
Yes	0.82 (0.78-0.88)	<0.0001	1.72 (1.55)

Most variables in three models are statistically significant except for Race, Marriage, UV exposure and Histological subtype. The HR of ulceration was drastically adjusted in model B and C, and it does not have significant difference between Model B and C. The E-value of Ulceration in Model B was 3.39. Most variables in Model B had a E-value higher than 3.39 or slightly less whereas other variables in Model C had a E-value less than 3. This result showed that variables other than MSAS were correctly taken out of the prediction model since they essentially did not affect the magnitude of effect of exposure (e.g. Ulceration) on MSS.

LL[1]=lower limit

Supplementary Table 6. HR of ulceration in training and validation set for model B and Harrell's C-index

		**Training set**	**Validation set**
HR	Yes/No	1.99	1.95
	95% CI	(1.88-2.09)	(1.81, 2.10)
	P-value	<0.0001	<0.0001
Harrell's C-index (SE)		0.887 (0.002)	0.887 (0.002)
Number of Observations		110029	55014

Both HR of ulceration and Harrell's C-index in the training and validation dataset indicate that the model was well fitted.

Reference

1. Sterne JA, White IR, Carlin JB, Spratt M, Royston P, Kenward MG, et al. Multiple imputation for missing data in epidemiological and clinical research: potential and pitfalls. Bmj. 2009;338.
2. Troyanskaya O, Cantor M, Sherlock G, Brown P, Hastie T, Tibshirani R, et al. Missing value estimation methods for DNA microarrays. Bioinformatics. 2001;17(6):520-5.
3. Schafer JL. Analysis of incomplete multivariate data: CRC press; 1997.
4. Van Buuren S, Oudshoorn K. Flexible multivariate imputation by MICE: Leiden: TNO; 1999.
5. Stekhoven DJ, Bühlmann P. MissForest—non-parametric missing value imputation for mixed-type data. Bioinformatics. 2012;28(1):112-8.
6. D'Agostino Jr RB. Propensity scores in cardiovascular research. Circulation. 2007;115(17):2340-3.
7. Ichino A, Mealli F, Nannicini T. From temporary help jobs to permanent employment: what can we learn from matching estimators and their sensitivity? Journal of applied econometrics. 2008;23(3):305-27.
8. Nannicini T. Simulation-based sensitivity analysis for matching estimators. The stata journal. 2007;7(3):334-50.
9. Becker SO, Ichino A. Estimation of average treatment effects based on propensity scores. The stata journal. 2002;2(4):358-77.
10. Caliendo M, Kopeinig S. Some practical guidance for the implementation of propensity score matching. Journal of economic surveys. 2008;22(1):31-72.
11. VanderWeele TJ, Ding P. Sensitivity analysis in observational research: introducing the E-value. Annals of internal medicine. 2017;167(4):268-74.
12. Royston P, Altman DG. External validation of a Cox prognostic model: principles and methods. BMC medical research methodology. 2013;13(1):1-15.
13. Thoemmes F, Rosseel Y, Textor J. Local fit evaluation of structural equation models using graphical criteria. Psychological methods. 2018;23(1):27.
